# Five-dimensional crystallography

**DOI:** 10.1107/S0108767309054166

**Published:** 2010-02-18

**Authors:** Marius Schmidt, Tim Graber, Robert Henning, Vukica Srajer

**Affiliations:** a Physics Department, University of Wisconsin-Milwaukee, Milwaukee, WI, USA; b BioCARS, Center for Advanced Radiation Sources, The University of Chicago, Chicago, IL, USA

**Keywords:** time-resolved crystallography, chemical kinetics, protein structure, temperature dependence

## Abstract

A method for determining a comprehensive chemical kinetic mechanism in macromolecular reactions is presented. The method is based on five-dimensional crystallography, where, in addition to space and time, temperature is also taken into consideration and an analysis based on singular value decomposition is applied. First results of such a time-resolved crystallographic study are presented. Temperature-dependent time-resolved X-ray diffraction measurements were conducted on the newly upgraded BioCARS 14-ID-B beamline at the Advanced Photon Source and aimed at elucidating a comprehensive kinetic mechanism of the photoactive yellow protein photocycle. Extensive time series of crystallographic data were collected at two temperatures, 293 K and 303 K. Relaxation times of the reaction extracted from these time series exhibit measurable differences for the two temperatures, hence demonstrating that five-dimensional crystallography is feasible.

## Abstract 2

Here it is demonstrated how five-dimensional crystallography can be used to determine a comprehensive chemical kinetic mechanism in concert with the atomic structures of transient intermediates that form and decay during the course of the reaction.

## 1. Introduction

### 1.1. Time-resolved crystallography

Time-resolved macromolecular crystallography is a unique method to unify chemical kinetics with X-ray structure determination (Moffat, 1998 ▶, 2001 ▶). Experiments are of the pump–probe type using ultra-short laser pulses as the pump to initiate a reaction in a protein crystal followed by a short and brilliant X-ray pulse of ∼100 ps duration to probe the structure at various time delays Δ*t*. This way, nanosecond and sub-nanosecond time resolution can be achieved to determine the kinetics of reactions in biological macromolecules as well as the structures of short-lived intermediates from the beginning of the reaction to the very end. Time-resolved crystallography has been used to investigate reactions in heme-containing proteins such as myoglobin (Srajer *et al.*, 1996 ▶, 2001 ▶; Schmidt *et al.*, 2005*a* ▶; Bourgeois *et al.*, 2003 ▶, 2006 ▶; Schotte *et al.*, 2003 ▶, 2004 ▶), FixL (Key *et al.*, 2007 ▶) and hemoglobin (Knapp *et al.*, 2006 ▶). In addition, the photocycle of the photoactive yellow protein was investigated extensively (Perman *et al.*, 1998 ▶; Ren *et al.*, 2001 ▶; Schmidt *et al.*, 2004 ▶; Rajagopal *et al.*, 2005 ▶; Ihee *et al.*, 2005 ▶) to determine the structures of the intermediates of its photocycle that form and decay conjointly with the chemical kinetic mechanism that connects them.

A major advance in extracting structures of intermediates and the kinetic mechanism from the time-dependent crystallographic data involved the application of singular value decomposition (SVD) to the time-resolved crystallographic data (Schmidt *et al.*, 2003 ▶). Application of the SVD-based global analysis made possible for the first time a theoretically sound analysis of the time-resolved X-ray data (Schmidt *et al.*, 2004 ▶; Rajagopal *et al.*, 2004 ▶, 2005 ▶; Ihee *et al.*, 2005 ▶). Although the structures of the intermediates can be determined objectively, a candidate kinetic mechanism is not unique. Multiple candidate mechanisms selected from a general mechanism (see below) can explain the data equally well. For a general understanding of protein function it is imperative to determine whether intermediates are on or off the catalytic pathway. Hence, the number of degenerate candidates must be reduced to only one unique mechanism. As a corollary, the general kinetic mechanism has to be covered comprehensively.

### 1.2. Chemical kinetics in time-resolved crystallography

**Figure 1:**
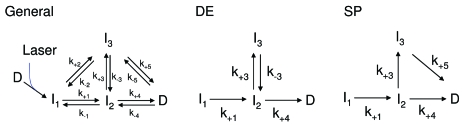
Kinetic mechanisms. General: general mechanism with three intermediates *I*_1_, *I*_2_ and *I*_3_, plus the reference (dark) state. The reaction is started by a laser pulse. DE: dead-end candidate, SP: semi-parallel candidate.

Chemical kinetics (Steinfeld *et al.*, 1989 ▶) describes a reaction in terms of schemes similar to the ones shown in Fig. 1 ▶, where a mechanism is depicted that employs only first-order reactions. Each step is characterized by its respective rate coefficient. Rate coefficients cannot be measured directly. Instead, macroscopic rates (or their negative inverse, the relaxation times) are directly observable in kinetic measurements. They are functions of all rate coefficients (Matsen & Franklin, 1950 ▶; Fleck, 1971 ▶). It is important to make a clear-cut distinction between experimentally observable (macroscopic) rates and the hidden (microscopic) rate coefficients of the mechanism (Rajcu *et al.*, 2009 ▶). It is the purpose and the goal of any kinetic experiment to ultimately determine the underlying kinetic mechanism with all of its rate coefficients. Almost any kinetic model is mathematically underdetermined if relaxation times from a time series at only one temperature are available, because a large number of rate coefficients must be determined from a smaller number of measured relaxation times. Since a time-resolved crystallographic experiment is a true kinetic experiment, it depends on the kinetic mechanism in fundamentally the same fashion.

**Figure 2:**
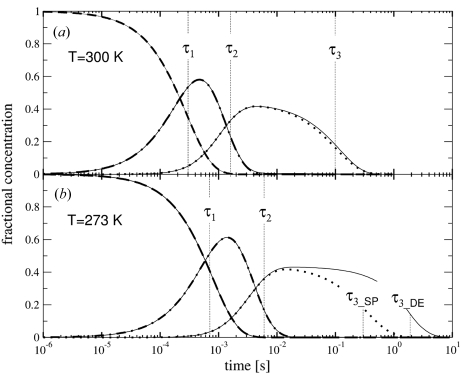
Concentration profiles and approximate relaxation times for the three intermediates in the semi-parallel and dead-end mechanisms. (*a*) 300 K, semi-parallel mechanism: intermediate *I*_1_ (dashed line), intermediate *I*_2_ (dashed dotted line) and intermediate *I*_3_ (dotted line). The thin lines represent the corresponding concentrations for the same intermediates in the dead-end mechanism. (*b*) 273 K, lines the same as in (*a*).

As an example we simulated relaxation times similar to those of a recent time-resolved crystallographic experiment (Schmidt *et al.*, 2004 ▶). Three relaxation times are observed. The number of relaxation times is equal to (or smaller than) the number of intermediates. A general chemical kinetic mechanism of a cyclic reaction with three intermediates plus the final (dark) state is shown in Fig. 1 ▶. The general mechanism contains 12 rate coefficients (that from *I*_1_ directly to *D* is not shown). It is immediately clear that the three relaxation times are not sufficient to uniquely determine all rate coefficients of the general mechanism. Therefore, two likely candidate mechanisms, each involving four rate coefficients, were picked in a rather subjective way (DE: dead-end candidate, SP: semi-parallel candidate). Fig. 2(*a*) ▶ shows that the rate coefficients for the two likely mechanisms can be such that the time-dependent concentrations of the intermediates match (almost) exactly at a certain temperature, here 300 K. At this temperature these two mechanisms are degenerate, because they give the same relaxation times. Now assume that in the DE mechanism intermediate *I*_3_ is very stable. That is, the barrier of activation to revert to intermediate *I*_2_ is quite high. Then, the rate coefficient *k*_−3_ becomes particularly small at lower temperatures. On the other hand, in the semi-parallel mechanism SP, intermediate 3 may branch directly to the final (dark) state, crossing a smaller energy barrier. If the temperature is lowered to, say, 273 K, these two mechanisms become separable (Fig. 2*b* ▶), although they were indistinguishable at 300 K. Consequently, the temperature adds observables that can be used to determine the unknowns (the rate coefficients) of the mechanisms.

The rate coefficients at two temperatures are related. In the simplest case this relationship is given by the Arrhenius equation (see, for example, Cornish-Bowden, 1999 ▶), where *k*_B_ is the Boltzmann constant and *T* is the temperature, 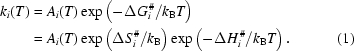
For each rate coefficient *k*_*i*_ we would need to determine three parameters: the enthalpy (Δ*H* ^#^) and entropy (Δ*S* ^#^) differences to the transition state, as well as a pre-factor *A*(*T*). In the simplest case, *A*(*T*) is proportional to the temperature (see also Cornish-Bowden, 1999 ▶). Hence, the fit would include a linear term, a constant and an exponential term. If we were able to determine experimentally only the relaxation times, we would need 12 different temperatures to account for the 36 free parameters in the general mechanism of this example. If we were only interested in the Δ*G* ^#^ values in equation (1), we would need eight temperatures (24 free parameters). The critical question is whether measurement over a limited physiological temperature range would allow us to determine all the unknowns from measured relaxation times.

However, we are in fact considerably better off, because we can exploit the so-called absolute scale present in crystallography. Measured structure factor amplitudes can always be placed on an absolute scale by scaling them to the calculated structure factor amplitudes from a precise known structural model. Electron density, when on an absolute scale, directly relates to occupancy; that is, to fractional concentration. For example, if a side chain has alternate conformations, the electron density is directly related to the occupancy of that conformation. The same is true for difference electron densities on the absolute scale. In contrast, in optical absorption spectroscopy, for example, absorption usually does not directly relate to concentration as there is a linear factor, the absorption coefficient, which is unknown *a priori* for various intermediates. In crystallography this linear factor is simply absent. Consider an experiment in which a CO molecule is photo-dissociated from the iron of a heme protein. By integrating the negative difference electron density at the CO binding site, seven electrons are obtained. Since CO has 14 electrons, 50% of the CO has been photo-dissociated. If the concentration of protein is 50 m*M* in the crystal, the remaining bound CO concentration is 25 m*M* (50% of the 50 m*M*). Note that this calculation is only valid on the absolute scale.

Now, we need to connect the structural result, the electron density, to the kinetics. Equation (2) is the result of integrating the coupled differential equations that describe the mechanism involving three intermediates assuming exponential kinetics. Equation (2) constitutes the mathematical base of simple chemical kinetics described in textbooks such as Steinfeld *et al.* (1989 ▶). The time-dependent concentrations [*I*_*j*_] can be calculated from the rate coefficients, because both the relaxation times τ_*k*_, which are the eigenvalues of the so-called coefficient matrix, and the pre-factors [*P*_*jk*_], which are the elements of the eigenvectors of the coefficient matrix, are functions of the rate coefficients; the [*P*_*jk*_] contain further specific initial conditions, for example [*I*_1_] = 1.0, [*I*_2_] = [*I*_3_] = 0 at *t* = 0; 
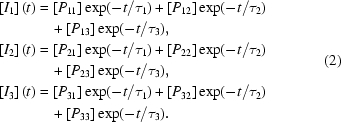
Now assume that we are able to observe the [*P*_*jk*_] directly. That would give us, in the best scenario, another nine independent observables to determine the underlying rate coefficients. However, some of the [*P*_*jk*_] could be very small, close to zero, because each intermediate might have only one transient. In our example in Fig. 2 ▶, [*P*_12_] and [*P*_13_] are in fact zero (and some others are also negligible in magnitude and not independent). This reduces the number of useful [*P*_*jk*_]. In any case, we would obtain at least three additional observables if the [*P*_*jk*_] could be observed. And indeed, the [*P*_*jk*_] can be observed directly in crystallography. How can that be done? This approach has been the foundation of the ‘posterior analysis’ developed by Schmidt *et al.* (2003 ▶, 2004 ▶) for post-SVD analysis of time-resolved data. Details are given also by Schmidt *et al.* (2005*b* ▶) and Schmidt (2008 ▶). In short, once the preliminary structures of the intermediates are determined, we can calculate time-independent difference electron densities for each intermediate by subtracting the structure factors of the known initial (reference) *D* state from those of the intermediates. We assume a chemical kinetic mechanism with initial values for the rate constants and generate with these calculated time-dependent difference electron densities 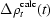 that can be compared directly with the observed difference densities 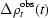. In a large fitting routine the rate coefficients and the initial condition, namely the concentration of activated molecules at the beginning of the reaction, are refined by minimizing globally the difference between the observed and calculated difference maps. Both the amplitudes [*P*_*jk*_] and the measured relaxation times τ_*j*_ are used as observables this way to refine the numerical values of the rate coefficients *k*_*i*_. The fact that concentration is directly related to electron density enhances greatly the ability to determine a comprehensive chemical kinetic mechanism from time-resolved crystallographic data.

With the relaxation times τ_*k*_ and the amplitudes [*P*_*jk*_] we gain at least six observables per temperature to determine the 36 free parameters (plus one initial condition, the extent of reaction initiation) in the general mechanism. That would reduce the number of required temperatures to only six or seven. It is extremely exciting to see that we actually have a realistic chance to determine a comprehensive general mechanism with measurements at a relatively small number of temperatures. However, the collection of an extensive spatially and temporally complete time series of Laue data is a tedious time-consuming process let alone the collection of time series at multiple temperatures. It is a major goal of this paper to show that this can now be achieved.

### 1.3. Data acquisition

BioCARS 14-ID-B is the only beamline in the USA dedicated to time-resolved macromolecular crystallography. It has been recently upgraded and features now the capability for 100 ps time resolution and an entirely new design to focus and deliver the X-rays to the sample. A single exposure with one 153 ps X-ray pulse in the hybrid mode of the APS storage ring can produce an acceptable Laue diffraction pattern. This dramatically speeds up data collection making the rapid collection of the entire time-series of Laue data feasible. In addition, it opens up new opportunities to investigate non-cyclic reactions in biomolecules. For the data presented here we used a nanosecond laser for reaction initiation; however, a picosecond laser system implemented at BioCARS by Philip Anfinrud and colleagues (NIDDK/NIH) is also available. It delivers 30 ps pulses to the sample and makes sub-ns time-resolution possible. New timing hardware and user-friendly software control modules enable fast automated data collection. To minimize radiation damage, larger crystals can be translated during data collection so that some fresh crystal volume is exposed each time to the intense X-ray pulses.

Here we show an optimized way to rapidly collect comprehensive spatially and temporally complete data sets from which the relaxation times of the kinetics can be extracted. We demonstrate how the relaxation times vary by changing the temperature by only 10 K, from 293 K to 303 K. These results will pave the way to a more comprehensive coverage of the available temperature range. Crystallography becomes five-dimensional, involving space, time and temperature, and with this the determination of a unique mechanism becomes feasible.

## 2. Material and methods

### 2.1. Data collection

**Figure 3:**
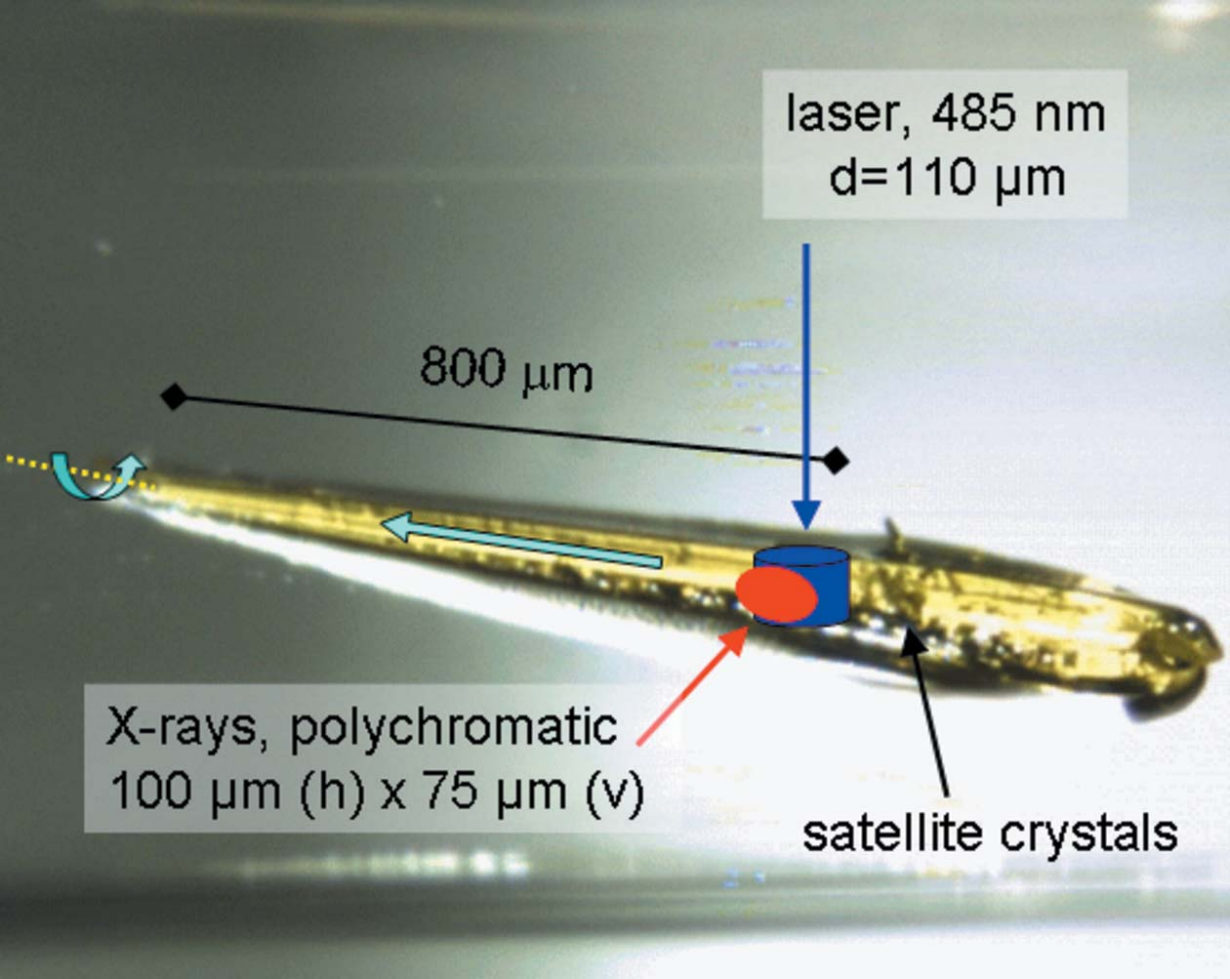
Typical laser and X-ray beam illumination geometry at the crystal. Needle-like PYP crystals of length >1 mm and diameter 80 µm were used. Only the region that was not compromised by the small satellite crystals was used for measurements. Arrows shown in cyan indicate translation and reorientation of the crystal during data collection.

Two comprehensive time series consisting of 28 Laue data sets (27 time-dependent data sets plus the reference dark data set) were collected on crystals of the photoactive yellow protein (PYP) at the BioCARS 14-ID beamline at the Advanced Photon Source, Argonne National Laboratory, Argonne IL, USA. One time series was collected at 293 K, the other at 303 K. For each time series only one long needle-shaped PYP crystal was used, each of dimensions 80 × 80 × 1000 µm. Crystals were mounted in glass capillaries of 1 mm diameter. X-ray beam size was 100 (h) × 75 (v) µm (Fig. 3 ▶). A reaction in the crystal was initiated by an intense laser pulse of 6 ns full width half-maximum (FWHM) at 485 nm (Vibrant Nd:YAG pumped OPO laser, Opotek), focused to a spot with a diameter of 110 µm. The total pulse energy was about 50 µJ which corresponds to an energy density of ∼5.3 mJ mm^−2^ at the crystal. Temperature was controlled by a cryojet sample cooler (Oxford Instruments). Data were collected with time as the fast variable (Rajagopal *et al.*, 2004 ▶). That means that at each crystal orientation data were collected at a series of time delays, then the crystal orientation was changed to a new value. The time delays were equidistant in logarithmic time, from 4 ns to 256 ms. For each time point, a Laue diffraction pattern was obtained by using seven (at 303 K) or eight (at 293 K) repeated 153 ps X-ray exposures, each preceded by a laser pulse. Based on previous experience (Schmidt *et al.*, 2004 ▶; Ihee *et al.*, 2005 ▶), the waiting time between the exposures was 2 s at 303 K and 4 s at 293 K. After all time points were completed at given crystal orientation, the crystal was rotated to collect data from another part of reciprocal space. The new orientation was selected in such a way that, if data collection would terminate prematurely, reciprocal space would have been covered in an approximately uniform manner. In order to reduce radiation damage, the crystal was also translated approximately 30 µm along its axis for each new crystal orientation. With a new setting all time points were again collected holding the crystal stationary. This was repeated until reciprocal space was covered by crystal settings a few degrees apart. The space group of PYP crystals is *P*6_3_. At 293 K, 24 different orientations were used to cover the unique volume of the reciprocal space with an angular spacing of 3°. At 303 K we used only 12 or 11 different settings with an angular spacing of about 6°. All diffraction images were collected on a Mar165 CCD detector.

### 2.2. Data reduction and difference maps

Laue data were indexed and integrated using *Precognition* and scaled using *Epinorm* (RenzResearch, http://www.renzresearch.com/). Table 1 ▶ shows the statistics of the comprehensive time series. The reference (dark) data were brought to the absolute scale by scaling them to structure factor amplitudes calculated from the PYP model 2phy from the Protein Data Bank (Berman *et al.*, 2000 ▶). The time-dependent Laue amplitudes were then scaled to their respective reference data. This way, both the reference data and the time-dependent data were on the absolute scale. Weighted difference structure factor amplitudes were calculated for each time point using 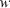 = 1/[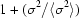 + 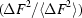] as weighting factors (see Ren *et al.*, 2001 ▶). Here, σ^2^ corresponds to an individual squared difference amplitude uncertainty relative to the mean square uncertainty found in the entire data set, 〈σ^2^〉. Δ*F* ^2^ is the squared difference amplitude and is compared with the mean square difference amplitude found in the entire data set 〈Δ*F* ^2^〉. This ensures that observations with large uncertainties and those with excessively high difference amplitudes are down-weighted for map calculation. Absolute scale is maintained by dividing (normalizing) the data by the average weighting factor. Difference maps were calculated with the weighted difference structure factor amplitudes using phases calculated from the reference model mentioned above.

### 2.3. Kinetic analysis

The time series of electron density maps were analyzed by singular value decomposition using the program *SVD4TX* (Schmidt *et al.*, 2003 ▶; Zhao & Schmidt, 2009 ▶). The SVD program masks out a given region in real space and performs an SVD on the region within the mask. The volumes occupied by the following residues were masked out: two different 4-hydroxycinnamoyl (HC4) chromophore structures found in the reference model and that of the pB2 photocycle intermediate (Schmidt *et al.*, 2004 ▶), Cys 69, Arg 52, Tyr42 and Glu46. These residues basically cover the chromophore binding pocket that contains the strongest signal ideal for this feasibility study. For a more comprehensive structural analysis, other parts of the PYP can also be masked out (Ihee *et al.*, 2005 ▶). The initial mask was evolved by only allowing grid points in the mask that contained difference electron density features larger than +3.0σ or smaller than −3.0σ that occur at least in one time point.

## 3. Results

### 3.1. Data collection efficiency and crystallographic quality

**Figure 4:**
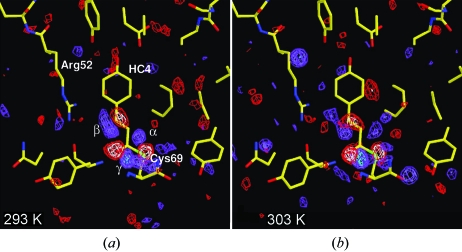
Difference electron density maps at a time delay Δ*t* of 256 ns at (*a*) 293 K and (*b*) 303 K. Contour levels: −3σ/−4σ (red/white); +3σ/+4σ/+5σ (blue/dark-blue/cyan). The atomic model displayed is that of the reference (dark) state (PDB 2phy structure) with the HC4-chromophore in its *trans* configuration. The electron density feature α shows the flip of the carbonyl oxygen of the HC4 tail and β shows the *trans* to *cis* isomerization about the double bond, while γ indicates the displacement of the Cys69 sulfur to which the chromophore is bound.

Owing to the high polychromatic X-ray flux at the sample at the 14-ID beamline, accumulation of only eight 153 ps X-ray pulses in the hybrid mode of the APS storage ring was sufficient to record very well exposed diffraction images. The total elapsed time for collecting the comprehensive time series at 293 K was about 6.5 h even with 4 s waiting time between the X-ray exposures. With only 2 s between the exposures an entire comprehensive time series can be collected in about 3.5 h. Using two fast Linux computers, data reduction of 56 PYP Laue data sets (28 data sets each at two different temperatures) can be completed in a few days using the *Precognition/Epinorm* software package. For crystals of other space groups that require covering a larger part of reciprocal-space data, data reduction time increases accordingly. Using 24 crystal orientations, PYP Laue data completeness is quite good to 1.8 Å and *R*_merge_ is around 7% throughout the entire resolution range, demonstrating the exquisite quality of crystallographic data obtained at 14-ID-B on relatively small diffracting volumes (80 × 75 × 100 µm) (Table 1*a* ▶). With 12 different crystal orientations, completeness is somewhat reduced (Table 1*b* ▶). However, the signal in the difference maps resulting from data collected with 12 orientations is comparable with that in the maps resulting from data with 24 crystal orientations as judged by the largest peak intensities of the difference features (Table 1 ▶ and Fig. 4 ▶). The data for the respective time series are remarkably homogeneous in terms of completeness and *R*_merge_. This is because each series was collected using only one crystal. The scale factors *R*_scale_ between the reference data and the time-dependent data for the last resolution shell increase slightly with each time point, indicative of some radiation damage (Table 1 ▶). However, the scale factors even at the highest dose do not substantially exceed those from scaling two reference data sets collected on different crystals [listed in the last columns of Tables 1(*a*) and 1(*b* ▶)]. This homogeneity of the data throughout the time series greatly contributes to the quality of the SVD analysis.

### 3.2. Radiation damage

**Figure 5:**
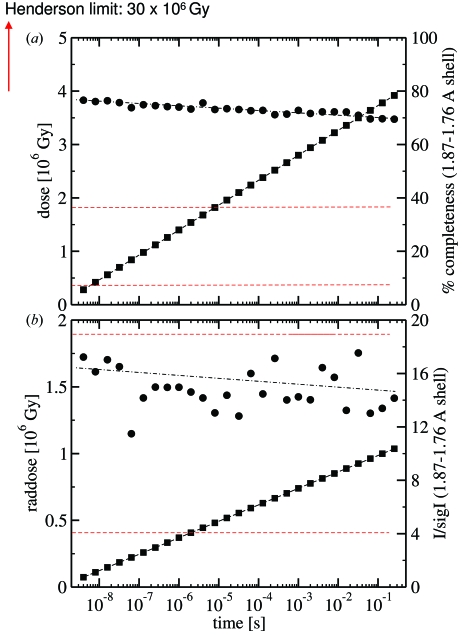
(*a*) Estimation of absorbed dose (full circles) and completeness (full squares) as a function of time. Since the time points were collected in consecutive order, dose becomes a function of time. Black dashed lines: guides to the eye. Red dashed lines: lower and higher dose rate limits. (*b*) Absorbed dose as calculated by program *Raddose* (full circles). *I*/σ_*I*_ (full squares) as a function of time is also shown. Red dashed lines: lower and higher dose rate limits.

The absorbed dose was calculated by estimating that roughly 3 × 10^10^ photons with an average energy of 12 keV are in a 153 ps X-ray pulse. Eight X-ray pulses were used to acquire a single CCD frame recording of a Laue diffraction pattern, containing a total of 2.4 × 10^11^ photons. With an X-ray beam size of 75 µm (v) × 100 µm (h), and crystal diameter of 80 µm, a crystal volume of 6 × 10^−7^ cm^3^ (beam size times crystal diameter) is exposed to these photons for each diffraction pattern (Fig. 3 ▶). As the translation of the crystal along the long crystal dimension (Fig. 3 ▶) before each new crystal orientation is 30 µm and the beam size is 100 µm, only one-third of fresh crystal volume is exposed to the X-rays each time the crystal is translated after the collection of 28 time points. This means that each diffracting crystal volume is actually exposed 3.3 times per time point. With a typical protein density of 1.35 g cm^−3^ (White *et al.*, 2007 ▶) the exposed volume contains about 810 ng of material. 2.4 × 10^11^ photons at 12 keV have a total energy of 0.46 mJ. Owen *et al.* (2006 ▶) give an approximate absorption coefficient of 1 mm^−1^ for a protein crystal. For the 80 µm PYP crystal, this means that 7.7% of the X-ray photons are absorbed and contribute to the absorbed dose given in Gray (Gy = J kg^−1^). Each volume along the needle-shaped crystal absorbs a dose of about 0.14 × 10^6^ Gy per time point (3.3 × 0.077 × 0.46 mJ/810 ng). In addition, the absorbed dose was also determined using the program *Raddose* (Murray *et al.*, 2004 ▶; Paithankar *et al.*, 2009 ▶). The absorption coefficient determined by *Raddose* was 0.22 mm^−1^. The absorbed photons generated a temperature jump of <0.1 K for each X-ray pulse. The accumulated dose determined from our crude estimation and that from *Raddose* are compared for all time points in Fig. 5 ▶.

### 3.3. Results from the SVD analysis

**Figure 6:**
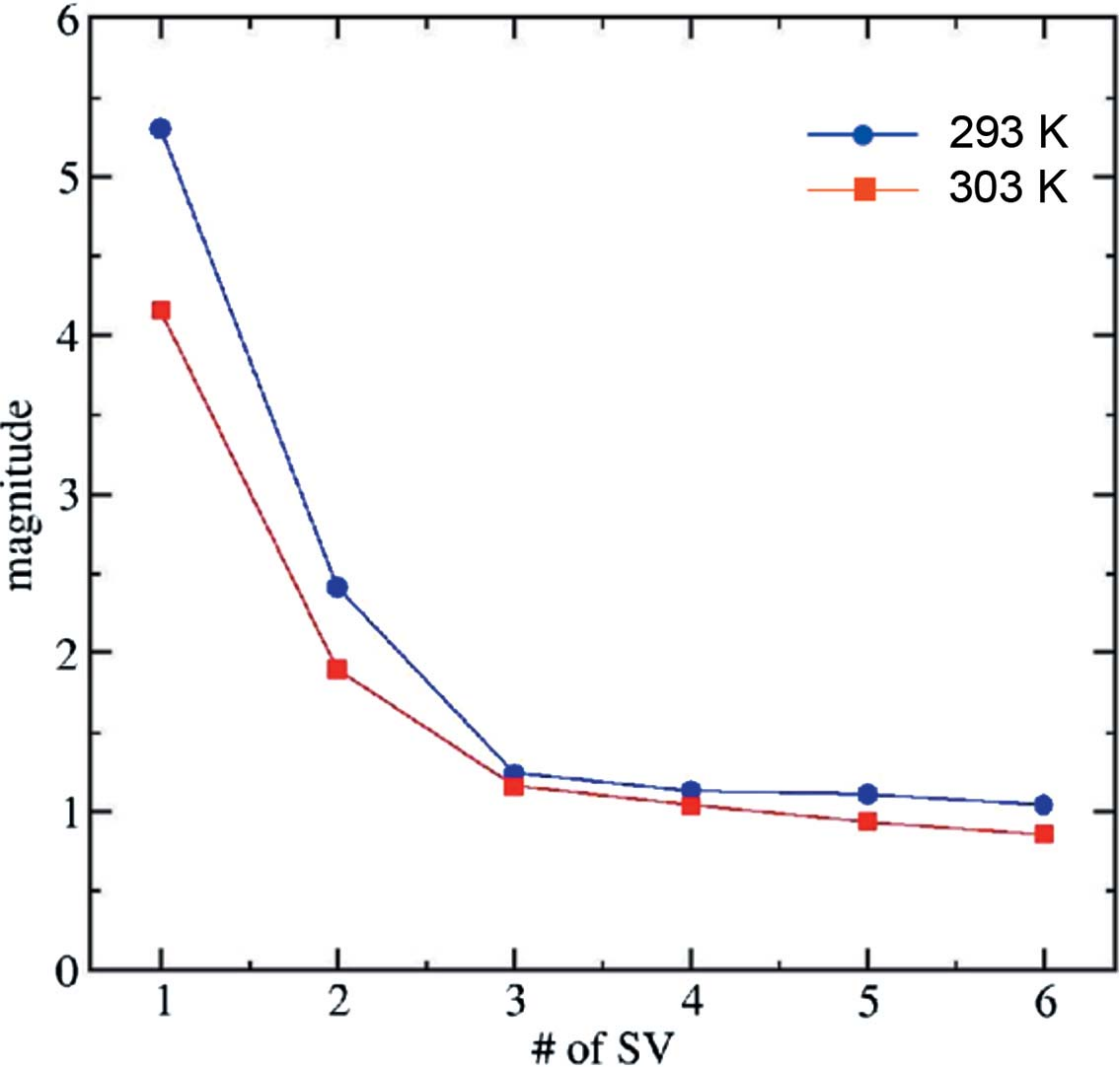
The first six (out of 27) singular values, SV, for the data at 293 K (blue) and 303 K (red). Two singular values are clearly significant.

**Figure 7:**
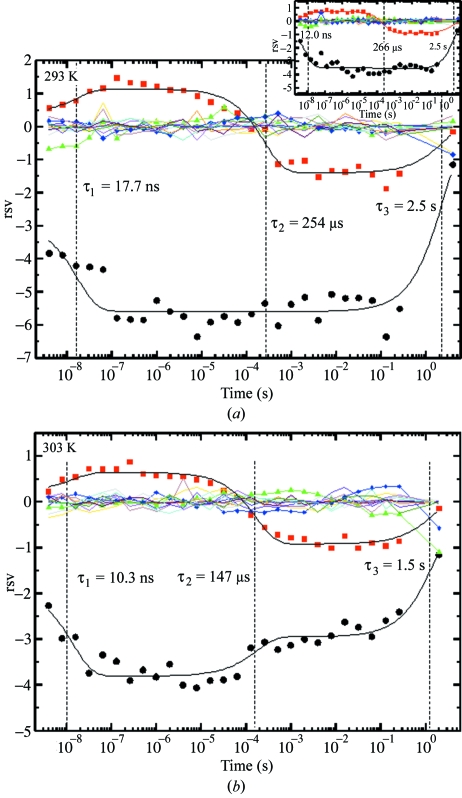
The right singular vectors (rsv) plotted as a function of time. Full circles: first significant singular vector; full squares: second significant singular vector. The third and fourth rsvs are shown by triangles and diamonds, respectively. Solid black lines through the first two significant rsvs are the result of a global fit using equation (3). (*a*) 293 K; insert: data set includes only 12 diffraction frames to match the number of frames in the 303 K data set. (*b*) 303 K.

The singular values and the right singular vectors (rsv) obtained from the SVD analysis of the time series of difference maps are shown in Figs. 6 ▶ and 7 ▶, respectively. Two obviously significant singular values are present (Fig. 6 ▶) at both temperatures. The rsv contain the kinetic information about the reaction. Those rsvs that belong to the two largest singular values are shown by circles and squares. Other, not so significant, rsvs are also shown in Fig. 7 ▶ by triangles and diamonds. These other rsvs might also contain some signal. However, since all relevant relaxation times are common to all significant rsvs, only the first two rsv_*i*_ (*i* = 1, 2) were analyzed here. These rsvs were globally fit by a sum of three exponential functions with relaxation times τ_1_, τ_2_ and τ_3_, 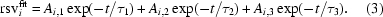
The pre-factors *A*_*i*,1_, *A*_*i*,2_ and *A*_*i*,3_ are (linear) fit parameters for each individual rsv, and the relaxation times τ_1_, τ_2_ and τ_3_ are common fit parameters for both rsvs. The result of the fit is shown in Fig. 7 ▶ by the solid lines through the data points of the first two rsvs. Table 2 ▶ shows the resulting values for relaxation times. All relaxation times are shorter at 303 K, indicating a faster reaction as expected.

## 4. Discussion

### 4.1. Radiation damage

The results presented here address the feasibility of five-dimensional crystallographic experiments and allow us to assess the suitability of such data for SVD-based global analysis of a kinetic mechanism. One of the major concerns is the radiation damage of the crystals owing to the total X-ray dose that is necessary for collecting a comprehensive time series of data (Figs. 5*a* and 5*b* ▶). Fig. 5 ▶ compares our estimation with the more accurate calculation by the program *Raddose* (Murray *et al.*, 2004 ▶; Paithankar *et al.*, 2009 ▶). Figs. 5(*a*) and 5(*b*) ▶ also plot the completeness as well as *I*/σ_*I*_ in the last resolution shell as a function of time. Since the time points are collected in consecutive order from one crystal setting, the crystals are exposed to a dose increasing by 0.14 × 10^6^ Gy per time point (see above) as estimated by our crude approximation. The dose calculated by *Raddose* was substantially smaller, only 0.037 × 10^6^ Gy per time point due to an about fourfold smaller absorption coefficient of 0.22 mm^−1^. At room temperature, crystal damage has been observed at 0.38 × 10^6^ Gy (Southworth-Davies *et al.*, 2007 ▶) suggesting that, between two and ten time points (dependent on the dose estimation), the crystals are damaged (lower dose rate limit). Interestingly, at room temperature the damage threshold *D*_1/2_ is largely dependent on, and increases with, the rate the dose is applied to the crystals (Southworth-Davies *et al.*, 2007 ▶). Even more, this effect is roughly independent on the elapsed time between the exposures. In our experiments 5300 Gy is absorbed in one single 153 ps X-ray pulse, which gives a dose rate of 3.5 × 10^13^ Gy s^−1^. Systematic investigations on radiation damage at these high dose rates do not exist so far. *D*_1/2_ values for dose rates of only 10 Gy s^−1^ are around 1.8 × 10^6^ Gy (higher dose rate limit in Fig. 5 ▶), suggesting a potential crystal damage after 12 time points (Fig. 5*a* ▶). The dose calculated by *Raddose* stayed well below the higher dose limit, so the damage seems to remain small (Fig. 5*b* ▶). The completeness in the last resolution shell of our PYP crystals as well as *I*/σ_*I*_ in the last resolution shell changes only slightly [Tables 1(*a*) and 1(*b*) ▶; Figs. 5(*a*) and 5(*b*) ▶], indicative of only a minimal effect of radiation damage. At cryogenic temperatures the radiation damage threshold is given by the Henderson limit which is around 3 × 10^7^ Gy (Henderson, 1990 ▶; Owen *et al.*, 2006 ▶), and of the order of 200 to 800 time points could be collected there. We believe that, owing to the extremely high dose rate, the dose threshold that limits the number of collectable Laue data sets is significantly higher than the high dose rate limit reported above and lower than the Henderson limit. At least with PYP, radiation damage seems to be small even after 28 time points. In addition, as we compare results for two crystals used in an identical fashion at two temperatures, any radiation damage should affect both crystals equally.

### 4.2. Limits

Another issue related to the success of five-dimensional crystallography is the ability to measure reliably relatively small changes in relaxation rates as a function of temperature. In the measurements presented here, the PYP HC4 chromophore clearly photo-isomerized from *trans* to *cis* as evident from the difference map shown in Fig. 4 ▶. The positive difference electron density features α and β are characteristic of such isomerization and represent one of the early intermediates in the PYP photocycle (Ihee *et al.*, 2005 ▶) that subsequently relaxes towards later intermediates (Schmidt *et al.*, 2004 ▶; Ihee *et al.*, 2005 ▶). The relaxation times of a reaction in general shift to shorter times if the temperature is increased. It is empirically observed that the reaction velocity doubles when the temperature is increased by 10 K (*Q*_10_ rule, see Cornish-Bowden, 1999 ▶). Here, we find that the second relaxation time τ_2_ changes by a factor of about 1.7, being 253 µs at 293 K and 147 µs at 303 K in good agreement with the *Q*_10_ rule. The change in the first relaxation time τ_1_ is more difficult to determine since the data are noisy in this time regime. However, the general trend that the relaxation times become shorter with increasing temperature can be observed also at the fast times (τ_1_ = 10 ns at 303 K and 17 ns at 293 K). The same trend is also observed if only 12 diffraction frames were merged at 293 K, to match the completeness of data collected at 303 K (Table 2 ▶, data in parentheses and Fig. 7 ▶, insert). This shows that the reduced reciprocal-space completeness at 303 K had no severe influence on the relaxation times. This is the first time that such a shift of relaxation times has been systematically observed for time-resolved crystallographic data. This was achieved by changing the temperature by only 10 K. The temperature range in which these experiments will be feasible spans from the freezing point of the liquor in the crystals which should be substantially below 273 K to the temperature where crystal stability is compromised by protein denaturation. PYP melts in solution at around 358 K (Meyer *et al.*, 2003 ▶). In crystals the melting temperature could be even larger. Hence, it should be possible to vary the temperature between 268 K and around 343 K. Seven to nine temperature settings separated by 10 K intervals will be achievable this way.

A comprehensive mechanism containing *N* states (*N* − 1 intermediates plus the ground state) can have *N*(*N* − 1) rate coefficients. Schmidt *et al.* (2003 ▶) determined the limits of the SVD-based analysis using three intermediates plus the ground state and five orders of magnitude in time covered by about three time points per logarithmic decade. Under those conditions, good results were obtained by the SVD analysis. With nine orders of magnitude in time covered, as presented here, at least five intermediates (*N* = 6) with 30 rate coefficients in the general mechanism could be accommodated. Incidentally, this is also the number of intermediates observed by Ihee *et al.* (2005 ▶). In the spirit of the above discussion, 90 free variables would face about ten observables per temperature point. For a comprehensive mechanism, nine different temperature settings would be necessary.

### 4.3. Effects of the laser that initiates the reaction

Empirical evidence shows that the excessive laser energy density at the sample induces increased transient and eventually permanent increase in crystal mosaicity as observed by elongation of diffraction spots, as well as gradual loss of diffraction at higher resolution. The pulse energy density we used is well below those density levels. In addition we used 4 ns laser pulses with a substantially lower peak power compared with the picosecond laser pulses that have also been used in the past for time-resolved experiments (Schotte *et al.*, 2003 ▶, 2004 ▶). Therefore, we believe that we avoided crystal damage by the laser pulses as much as possible.

A laser-induced temperature jump is also a potential challenge for five-dimensional crystallography. For the data presented here, a laser beam with a 110 µm diameter delivered about 90% of the total pulse energy or ∼45 µJ into the crystal volume of 110 × 80 × 80 µm. Using the typical crystal density of 1.35 g cm^−3^ given above, this volume contains 9.5 × 10^−10^ kg. The heat capacity of protein crystals is of the order of 3 to 8 kJ kg^−1^ K^−1^ (Miyazaki *et al.*, 1993 ▶, 2000 ▶). Assuming that all of the laser pulse energy delivered to the crystal is absorbed within the crystal volume above (which is consistent with the optical density of the crystal at 485 nm), the temperature jump in the crystal generated by the laser pulse ranges from 6 to 16 K. Thermal diffusion times in the crystal are of the order of 50 ms (Moffat *et al.*, 1992 ▶). We can expect that on the fast time scale, up to a few tens of milliseconds, the same temperature offset owing to laser-induced heating will be present at all temperatures. However, special attention has to be given to the slower time scales. The time-dependent temperature profile in the crystal should be initially modelled based on crystal and laser beam geometry as well as taking laser power, heat capacity and heat conductance in protein crystals into account (Noelting, 1998 ▶). This profile can be likely refined using the five-dimensional crystallographic data at later stages of the analysis.

### 4.4. Mechanisms

In recent years, a number of papers have reported effects on kinetic variables owing to molecular crowding that occurs under physiological conditions in the cell (see Zhou *et al.*, 2008 ▶ for a recent review). Binding constants and reaction rates are altered compared with dilute solution. Protein crystals are ultimately crowded. It is not clear whether crystals or dilute solutions best resemble the situation *in vivo*.

Finally, it has been observed that the simple exponential approach to reaction kinetics fails at cryogenic temperatures where the time scales of conformational fluctuation are drastically reduced or frozen, leading to an ensemble of molecules with a distribution of rate coefficients (Austin *et al.*, 1975 ▶). Since time-resolved crystallographic experiments are performed at ambient temperatures, the protein molecules are flexible (Parak, 2003 ▶; Schmidt *et al.*, 2009 ▶) and the ensemble likely obeys classical chemical kinetics (Tetreau *et al.*, 2004 ▶) as long as the rate of conformational averaging is fast compared with the reaction rates.

## Figures and Tables

**(a):** Every second time point is listed. 24 crystal orientations were used to cover the unique volume of reciprocal space. *R*_merge_ is given for the entire resolution range to 1.6 Å. *R*_scale_ is the result from scaling time-dependent and dark (reference) data sets to a resolution of 1.76 Å. Difference maps were calculated to 1.8 Å.

			Δ*t* (light)														
		Dark	4 ns	16 ns	64 ns	256 ns	1 µs	4 µs	16 µs	64 µs	256 µs	1 ms	4 ms	16 ms	64 ms	256 ms	4 s†
Completeness, ∞–1.76 Å (%)		89.2	89.2	88.9	88.3	88.3	87.9	87.9	87.6	87.3	87.6	87.3	87.4	87.1	86.6	86.4	
Completeness, ∞–3.5 Å (%)		91.2	91.4	91.3	91.3	92.0	91.4	91.4	91.5	91.2	91.2	91.6	91.1	91.2	91.6	91.6	
Last shell		78.4	76.9	76.6	73.8	74.6	74.0	74.6	73.4	73.9	71.3	74.9	72.3	72.2	69.7	69.6	
*I*/σ*I*		18.0	17.2	17.0	11.5	15.0	15.0	14.2	14.4	16.0	17.1	14.4	16.4	13.2	13.0	14.1	
*R*_merge_ on |*F*| (%)		3.6	4.2	4.4	6.4	4.5	4.7	4.8	4.9	4.8	4.4	4.7	4.8	4.8	4.9	5.0	
*R*_merge_ on |*F*|^2^ (%)		6.4	7.4	7.7	11.1	7.8	8.8	8.4	8.5	8.5	7.6	8.3	8.5	8.4	8.4	8.8	
*R*_scale_ (%)‡			8.8	9.1	11.4	10.6	10.6	11.0	11.6	10.9	11.3	12.2	11.9	11.4	11.8	13.4	12.1
〈Δ*F*〉 (e^−^)			12.1	13.7	20.4	15.1	16.8	17.0	17.6	17.7	16.9	17.6	17.7	18.5	11.8	20.1	25.5
(σ_Δ*F*_)			3.7	4.1	4.5	4.1	4.3	4.4	4.5	4.5	4.5	4.5	4.4	4.7	4.8	4.8	4.1
Δρ_max/min_/σ_Δρ_§			−7.6	−7.0	−5.0	−7.0	−6.4	−6.7	−6.0	−6.0	−4.7	−4.5	−5.1	−5.0	−5.1	−4.9	−4.6
			+6.0	+5.0	+5.0	+6.2	+6.1	+5.6	+5.4	+4.8	+4.7	+4.5	+5.1	+4.5	+4.8	+4.5	+4.5

**(b):** Every second time point is listed. Up to 32 µs 12 crystal orientations and for larger time delays 11 crystal settings were used to cover the unique volume of the reciprocal space. *R*_scale_ is the result from scaling time-dependent and dark (reference) data sets to a resolution of 1.76 Å. Difference maps were calculated to 1.8 Å.

			Δ*t* (light)														
		Dark	4 ns	16 ns	64 ns	256 ns	1 µs	4 µs	16 µs	64 µs	256 µs	1 ms	4 ms	16 ms	64 ms	256 ms	2 s¶
Completeness, ∞–1.76 Å (%)		70.7	70.4	69.8	69.9	69.5	69.6	68.8	68.9	65.7	65.7	65.7	65.3	65.1	64.9	65.0	
Completeness, ∞–3.5 Å (%)		74.3	74.0	74.2	74.2	74.3	73.9	72.2	73.7	73.5	71.9	71.9	71.8	71.4	71.6	72.0	
Last shell		52.7	51.2	51.2	50.8	49.9	50.6	48.4	48.3	48.5	45.3	45.6	43.9	44.0	44.3	43.9	
*R*_merge_ on |*F*| (%)		3.2	3.5	3.4	3.6	3.5	3.9	3.8	3.8	3.8	3.8	3.8	3.9	3.8	3.8	3.9	
*R*_merge_ on |*F*|^2^ (%)		5.7	6.1	6.0	6.4	6.2	6.9	6.7	6.6	6.6	6.8	6.8	6.9	6.6	6.7	6.9	
*R*_scale_ (%)††			9.3	9.6	11.0	10.4	11.3	11.3	10.9	11.9	11.3	12.9	12.0	11.4	12.4	13.3	12.2
〈Δ*F*〉 (e^−^)			11.4	12.9	15.0	14.7	15.9	16.7	15.7	16.8	17.1	18.0	18.7	18.0	18.4	18.9	25.2
〈σ_Δ*F*_〉			4.5	4.6	4.8	4.8	4.9	4.9	5.1	5.1	5.2	5.3	5.4	5.5	5.5	5.7	4.1
Δρ_max/min_/σ_Δρ_‡‡			−5.5	−5.9	−6.4	−6.1	−5.1	−6.5	−6.2	−4.5	−4.3	−4.5	−4.5	−4.7	−4.5	−4.8	−4.3
			+4.4	+6.2	+5.8	+8.1	+6.1	+5.1	+5.7	+4.5	+4.3	+4.5	+4.7	+4.4	+4.5	+4.6	+4.5

† For the 4 s time point the reference (dark) data at 303 K were subtracted from the reference (dark) data collected at 293 K.

‡ *R*_scale_ given here is for the last shell: 1.87–1.76 Å.

§ Highest positive and lowest negative difference electron density feature (Δρ), in units of σ value of the difference map.

¶ For the 2 s time point the reference (dark) data at 293 K were subtracted from the reference (dark) data collected at 303 K.

†† *R*_scale_ given here is for the last shell: 1.87–1.76 Å

‡‡ Highest positive and lowest negative difference electron density feature (Δρ), in units of σ value of the difference map.

**Table 2:** Relaxation times Numbers in parentheses are for data at 293 K where only 12 frames were merged to match the number of frames in the 303 K data set.

*T*	τ_1_	τ_2_	τ_3_
293 K	17.7 ns (12.0 ns)	254 µs (266 µs)	2.5 s (2.5 s)
303 K	10.3 ns	147 µs	1.4 s
